# ANGPTL3 is involved in kidney injury in high-fat diet-fed mice by suppressing ACTN4 expression

**DOI:** 10.1186/s12944-022-01700-3

**Published:** 2022-09-19

**Authors:** Guanyu Li, Di Lu, Jingzhi Wang, Shuling Yue, Mei Tan, Ming Liu, Xia Gao

**Affiliations:** 1 Nephrology Department, Guangzhou Women and Children’s Medical Center, Guangdong Provincial Clinical Research Center for Child Health, Guangzhou Medical University, No. 318 Renmin Middle Road, Guangzhou City, 510623 China; 2 Guangzhou KingMed Diagnostics Group, Guangzhou City, 510623 China; 3 Guangdong Provincial Clinical Research Center for Child Health, Guangzhou Institute of Pediatrics, Guangzhou Women and Children’s Medical Center, Guangzhou City, 510623 China

**Keywords:** ANGPTL3, ACTN4, Podocin, Podocyte injury, Hyperlipidemia renal injury

## Abstract

**Objective:**

We wanted to explore how angiopoietin-like 3 (ANGPTL3) impact hyperlipidemia-induced renal injury.

**Methods:**

ANGPTL3 knockout mice and wild-type C57 mice were set up in four groups (*N* = 5) depending on a normal or 60% high-fat diet: wild-type with normal diet (WT), *angptl3*-/- with normal diet (KO), wild-type + high-fat diet (WT + HF) and *angptl3*-/- + high-fat diet (KO + HF). The detection time points were the 9th, 13th, 17th and 21st weeks after modeling. Serum lipid and urinary protein levels of mice in each group were detected, and pathological changes in the kidney were analyzed. Moreover, the expression of ANGPTL3, α-actinin-4 (ACTN4), CD2-associated protein (CD2AP) and podocin was tested in the glomerulus by immunohistochemistry (IHC).

**Results:**

In the WT + HF group, hyperlipidemia and proteinuria could be observed at the 9th week and were gradually aggravated with time. Compared with WT + HF mice, the levels of serum lipids and proteinuria in KO + HF mice were significantly reduced, and the width of podocyte foot processes (FPs) fusion was also markedly improved. The IHC results suggested that in WT + HF mice, the expression of ANGPTL3 was significantly enhanced. After modeling, ACTN4 expression was markedly weakened in the glomeruli of WT + HF mice. Different to WT mice, ACTN4 expression was not observed obviously change in KO + HF mice. Compared with the normal diet group, the expression of podocin showed a decline in WT mice treated with high-fat diet and showed a significant difference from the 17th week. In addition, podocin expression in KO + HF glomeruli was also found to be weak but not significantly different from that in WT + HF glomeruli at the four time points. The expression of CD2AP showed similar results among the four groups.

**Conclusion:**

ANGPTL3 could play a role in the mechanism of hyperlipidemia-associated podocyte injury via ACTN4.

**Supplementary Information:**

The online version contains supplementary material available at 10.1186/s12944-022-01700-3.

## Introduction

Despite decades of widespread attention on the relationship between dyslipidemia and chronic kidney disease (CKD), the mechanism of hyperlipidemia leading to CKD has not yet been elucidated. Clinically, there is still a lack of targeted drugs to prevent the appearance of renal injury with hyperlipidemia.

Apolipoprotein APOA1 and APOB were first discovered in the kidney, revealing that the kidney is also a source of lipoprotein secretion [1]. Recent reports have confirmed that lipoprotein lipase (LPL) can be secreted by glomerular mesangial cells [2]. Podocytes have also been reported to express apolipoprotein L1, an important component of HDL, whose gene polymorphism is associated with focal segmental glomerulosclerosis (FSGS).

Angiopoietin-like 3 (ANGPTL3) is a member of the angiopoietin family and mainly expressed in hepatology cells. Now, ANGPTL3 is regarded as a key factor in lipid metabolism regulation. The main functions of ANGPTL3 in lipid metabolism include the following: 1. Inhibiting LPL activity, which can reduce the clearance rate of triglycerides (TG) and increase the level of plasma triglycerides. 2. Inhibiting endothelial lipase phospholipase activity, which leads to decreased plasma high-density lipoprotein hydrolysis; 3. Accelerating the transfer of fatty glycerol and fatty acids from adipocytes to the liver after specifically binding to adipocytes. Because the further transformation products of these substrates in the liver are triglycerides and glucose, they will increase the content of free fatty acids in plasma [3–5].

Recently, study found that the serum and urine ANGPTL3 levels of patients with hyperlipidemia-induced renal injury remarkedly enhanced compared to those of healthy controls. Among them, serum level of ANGPTL3 was positively correlated with the patients’ lipid indexes and 24hPro level [6]. Based on these results, it seems that ANGPTL3 contributes to the development of hyperlipidemic proteinuria. Recent studies on ANGPTL3 and podocyte injury have revealed that this molecule binds to the integrin β3 receptor on the surface of podocytes and then activates the PI3K/ACTN4 signaling pathway, resulting in rearrangement of the cytoskeleton and an increase in podocyte activity. The rearrangement of the cytoskeleton in vitro is the molecular basis of podocyte FPs fusion in vivo [7, 8]. In addition, in a study of the podocyte slit diaphragm, a series of endogenous molecules have been found, which have a special relationship with podocyte function. These endogenous molecules include transient signal regulatory proteins and CD2-associated proteins. In addition, NPHS2 and the structural protein α-actinin-4 are also included [9].

The goal of the study was to explore the relationship between ANGPTL3 and podocyte injury when mice developed hyperlipidemic kidney injury using *angptl3* gene knockout mice and its potential role in the expression of podocyte lipid raft-associated molecules, including ACTN4, podocin and CD2AP.

## Materials and methods

### Grouping of experimental animals

A total of 40 *angptl3* knockout mice (*C57BL/6 J* background) and 40 wild-type *C57BL/6 J* mice, which were 6 weeks old, were raised in a specific pathogen-free (SPF) laboratory. All animal experiments were approved by the animal welfare and ethics committee of Gansu Provincial People’s Hospital (No: syll20160037). The animal center at Gansu University of Traditional Chinese Medicine provided a place for the mice. A normal diet (10% fat, 20% protein, and 70% carbohydrates) and a high-fat diet (American Research Diets, 70% fat, 15% protein, and 25% carbohydrates) were administered to the model mice randomly at the beginning of the experiment. Wild-type mice were set up two groups, normal diet (WT) and high-fat diet (WT + HF). *Angptl3* knockout mice were also set up two groups, normal diet (KO) and high-fat diet (KO + HF). All animals had plenty of food, and the frequency of feed and drinking water changes was three times a week. Mice were euthanized at weeks 9, 13, 17, and 21 post-modeling, with 5 mice per group.

### Method of retaining samples

Before sacrificing the mice, they were weighed, and 24-h urine was collected with metabolic cages. To obtain serum, samples were collected from the retrobulbar vein and centrifuged for 15 min at 800 × g. Urine and serum were stored at -80 °C. Kidneys were harvested and rinsed with PBS, and capsule was removed. Half of kidney was placed in a -80 °C freezer, and the other half was used for histological evaluation. Tissue was embedded in paraffin after fixation with 4% paraformaldehyde, and sections were stained using immunohistochemistry to assess renal injury. Renal cortical tissue of 1 cubic millimeter was taken and fixed with 2.5% glutaraldehyde and then evaluated using transmission electron microscopy.

### Detection of urine protein, triglyceride and cholesterol (TC)

The detection of urinary protein, TG and TC concentrations was carried out at the Clinical Laboratory Center. The automatic biochemical analyzer was provided by the people's Hospital of Gansu Province (ISO15189 Medical Laboratory Accreditation in 2015).

### Immunohistochemistry

The experimental method was as described previously [10]. The primary antibodies were angiopoietin-like 3 polyclonal antibody (Abcam, Cambridge, UK, ab126718), ACTN4 monoclonal antibody (Abcam, Cambridge, UK, ab108198), podocin antibody (Abcam, Cambridge, UK, ab229037) and anti-CD2AP antibody (Sigma‒Aldrich, MO, USA, HPA003267). The secondary antibody was horseradish peroxidase-labeled goat anti-rabbit IgG (Immunoway, Plano, USA, RS0002).

The analysis method refers to a previous article [10]. In brief, the IHC results were analyzed semiquantitatively using ImageJ 1.50. Positive area and positive intensity values in glomeruli were measured in IHC-stained sections, and the IHC index was equal to the positive area multiplied by the intensity value. The IHC index was used to determine the levels of ANGPTL3, ACTN4, podocin and CD2AP.

### Statistical analysis

Measurement data were analyzed using a *t* test (two-tailed) to compare the two groups. The method used to compare multiple groups was one-way ANOVA. The experiment was repeated three times. A test value of *P* less than 0.05 was considered significantly different. * *P* < 0.05; ** *P* < 0.01.

## Results

### High-fat diet could be due to hyperlipidemia and proteinuria in mice

**Fig. 1:**
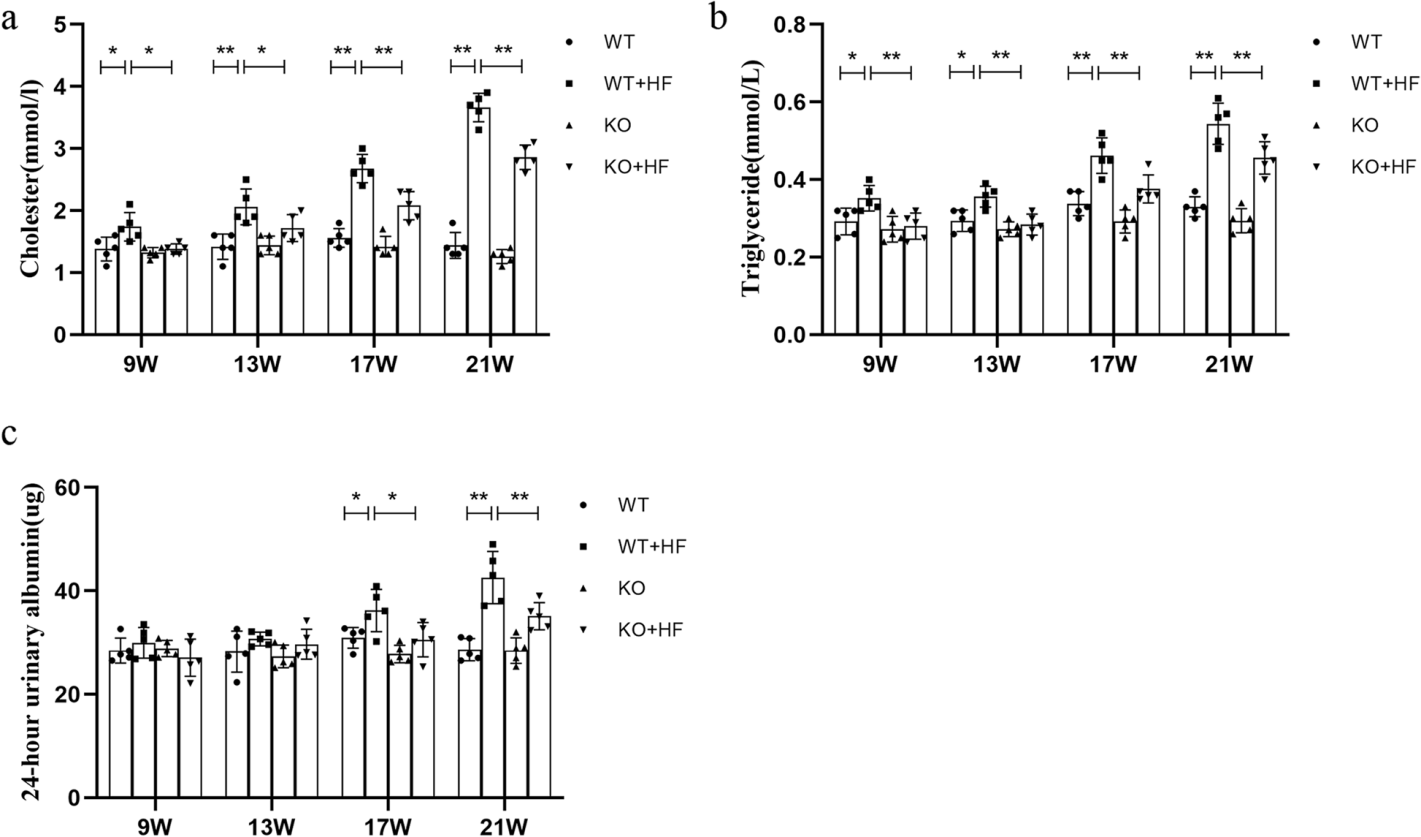
Serum lipid index and proteinuria levels in the four groups. **a, b** In the WT + HF group, the levels of TG and TC were significantly increased, and both levels were significantly lower in the KO + HF group than in the WT + HF group. c: The levels of 24-h urinary albumin increased in the WT + HF group, and the proteinuria level was relieved in the KO + HF group

At the first detection point after modeling, serum TG and TC concentrations were noteworthily more in the WT + HF mice than in the WT mice (*P* < 0.05). As shown in Fig. 1a, b, with prolonged modeling time, the increases in TG and TC were gradually significant in the WT + HF mice. Further analysis of 24-h urinary albumin showed that urinary albumin in the WT + HF mice was enhanced compared with that in the WT mice and showed a significant difference at the 17th week (Fig. 1c).

### Podocyte injury was observed in the WT + HF group

**Fig. 2:**
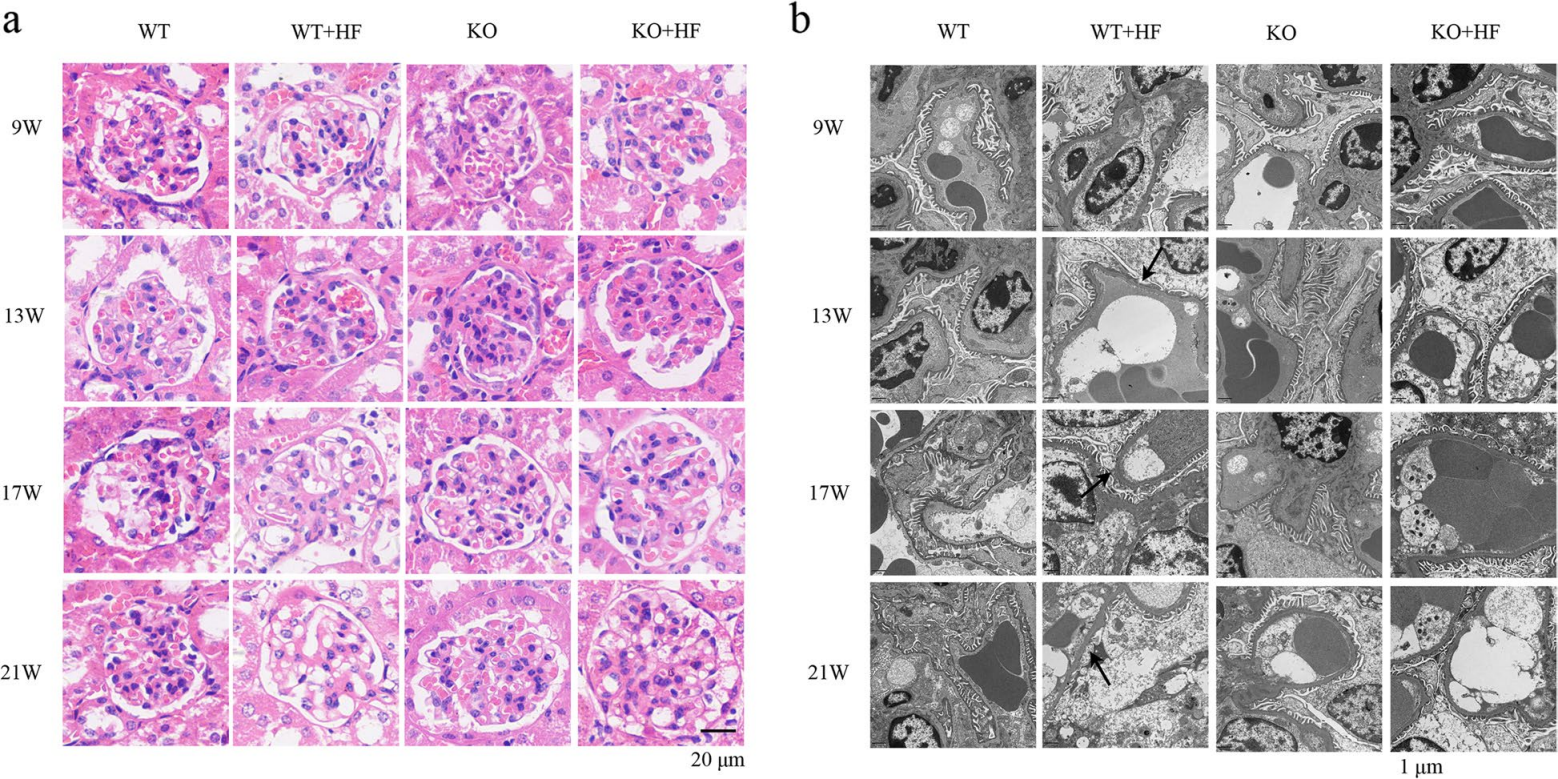
The kidney pathologic differences in the four groups. **a** HE staining showed no obvious changes in any group. (Bars = 20 μm) **b**. The electron microscope showed podocyte FP fusion and disappearance in the WT + HF group (arrow). And the fusion degree was alleviated in the KO + HF group (Bars = 1 μm)

The H&E staining results are shown in Fig. 2. No glomerulosclerosis or segmental sclerosis was seen on renal histomorphology. There was no crescentic fibrosis present in Bowman’s space (Fig. 2a). Under the electron microscope, as shown in Fig. 2b, after the first 9 weeks of feeding, slight fusion of podocyte FPs appeared in the WT + HF group. At the 17th week, the degree of podocyte FP fusion was further aggravated. A small amount of electron dense deposition and thickening of the basement membrane were observed in the mesangial area in the WT + HF group.

### Lack of ANGPTL3 could not impaired the renal and lipid metabolism function of the mice under physiological conditions

*angptl3* knockout mice (Supplementary Fig. 1) were established using CRISPR/Cas9 technology according to a previously published article [11]. As shown in Fig. 1a, b, serum TC and TG decreased in the KO mice than in the WT mice at each observation time point; however, no significant difference was observed between the groups, which was not consistent with previously reported data [8]. In this study, 24-h urinary albumin was used as the main evaluation index of renal injury in mice. Under normal feeding conditions, the level of urinary albumin in the KO mice was similar to that in the wild-type mice (Fig. 1c).

Correspondingly, the glomeruli of *angptl3* knockout mice were basically normal, with no inflammatory cell infiltration or fibrous crescent formation (Fig. 2a). Under transmission electron microscopy, no abnormalities were observed in the podocytes or basement membrane (Fig. 2b).

### Expression of ANGPTL3 was markedly augmented after high-fat feeding

**Fig. 3:**
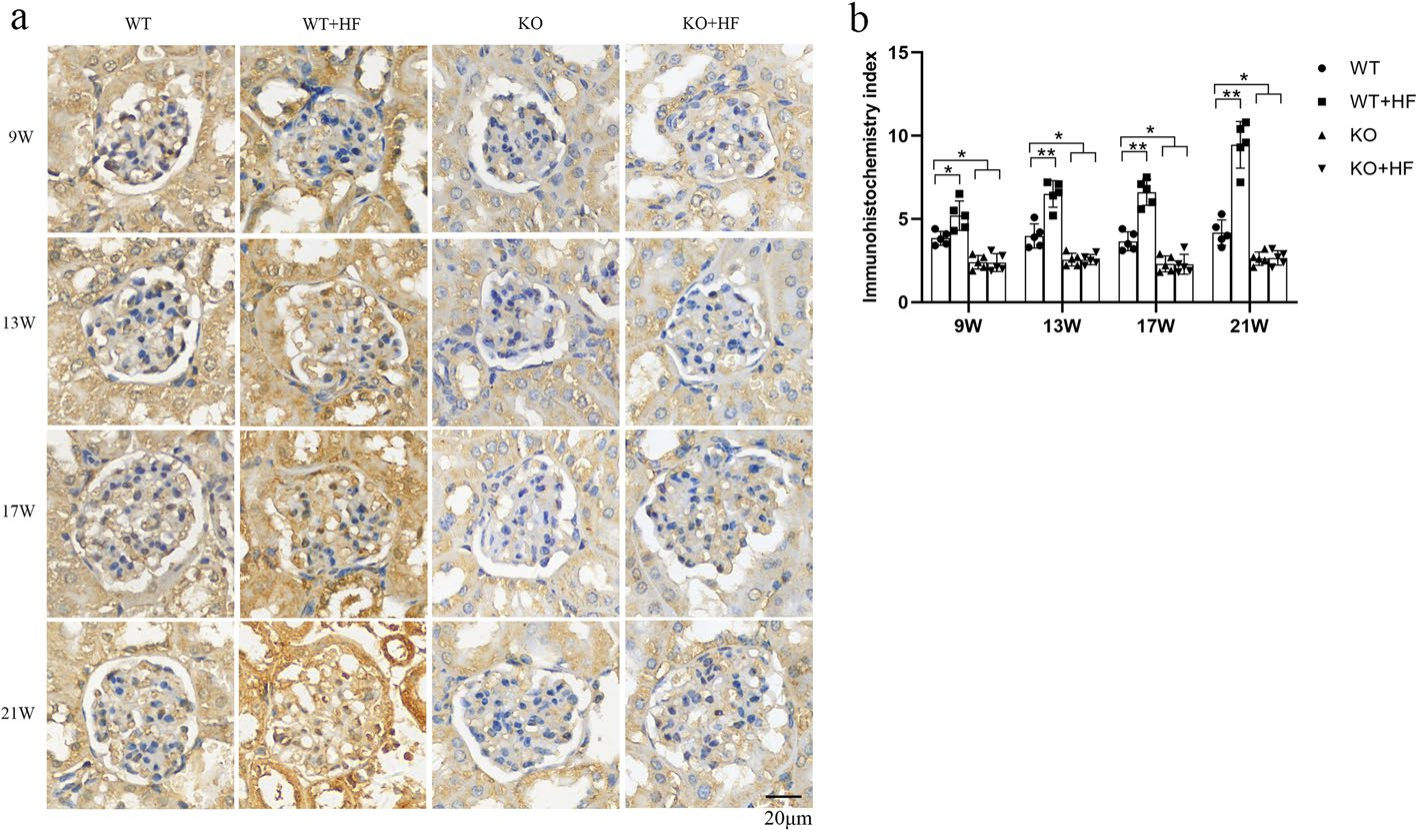
Immunohistochemistry of ANGPTL3 in mouse renal tissue. **a** At the first time point, the ANGPTL3 staining in the glomerulus along the loops of the blood vessels was significantly strengthened in the WT + HF group. **b** Compared with the WT group, the IHC intensity scores of ANGPTL3 in the WT + HF group were significantly different at each time point. (Bars = 20 μm)

It is reported that podocyte could weakly synthesis ANGPTL3 [8]. In this study, the IHC results in glomeruli of the WT group showed that the expression of ANGPTL3 was weak. From the first observed time point after modeling, ANGPTL3 in WT + HF glomeruli was gradually enhanced and distributed along the vascular ring of the glomerulus (Fig. 3a). The IHC intensity scores showed that ANGPTL3 expression was powerfully stronger in the WT + HF glomerulus than in the WT glomerulus (Fig. 3b).

### The mice knocked-out *angptl3* gene showed a resistance to high-fat diet induced- renal injury and lipid dysfunction

The phenotypes of *angptl3* knockout mice and wild-type mice are phenotypically different in the high-fat feeding status. First, the TG levels in KO + HF mice gradually increased and showed significant differences from those in KO mice at the third and fourth observation points. The levels of TC in the KO + HF mice showed differences from the KO mice starting at week 13. However, statistics showed that *angptl3* knockout mice had significantly improved lipid metabolism indices compared to those of WT mice, especially in the high-fat feeding setting. As shown in Fig. 1, for all four observation time points, the KO + HF group plasma TG levels were notedly declined than those of the WT + HF group (Fig. 1a, b), and the significance became more pronounced with a longer observation time. At all four time points, the 24-h urinary albumin results showed that urine albumin levels were lower in the KO + HF group than in the WT + HF group, with statistically significant differences at weeks 17 and 21 (Fig. 1c). In the WT + HF group, the podocyte structure was basically normal at 9 and 13 weeks, and at the 17th and 21st weeks, the podocyte foot processes were effaced (Fig. 2b).

### Lack of ANGPTL3 inhibited the decreased expression of ACTN4 in podocytes after high-fat feeding

**Fig. 4:**
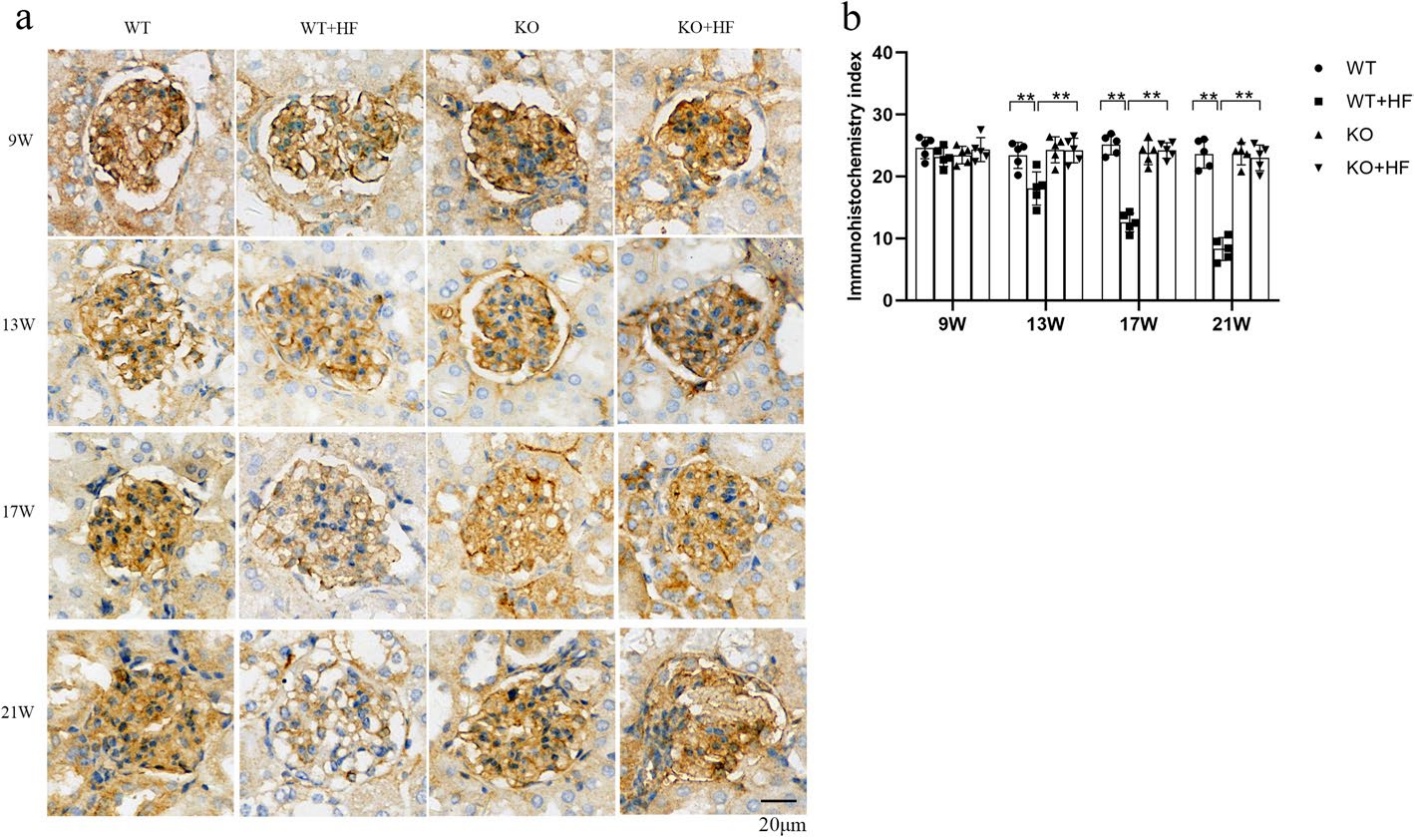
Lack of ANGPTL3 inhibited the decreased expression of ACTN4 in podocytes after high-fat feeding. **a** The staining intensity of ACTN4 is shown in the four groups; **b** the staining intensity gradually decreased in the WT + HF group compared to the WT group. In contrast, the staining intensity did not decline in KO + HF mice. (Bars = 20 μm)

ACTN4 is a podocyte skeletal regulatory protein. High expression or the absence of ACTN4 can lead to pathological phenomena such as increased podocyte activity, FPs fusion and podocyte detachment. ACTN4 staining was mainly distributed in the capillarity network of blood vessels within the glomeruli. In the WT + HF group, ACTN4 staining intensity showed a diminishing trend (Fig. 4a). After the 13th week, compared with that in the WT group, the staining intensity in the WT + HF group began to decrease (Fig. 4b).

Under normal conditions, the IHC data suggested that ACTN4 express in the glomerular was not altered by *angptl3* knockdown (Fig. 4a). The ACTN4 staining intensity in the KO + HF group remained strong throughout the whole time period and was closer to that in the WT group. Furthermore, the difference between WT + HF and KO + HF showed statistical value (Fig. 4b).

### ANGPTL3 did not impact the podocin decline in podocytes in hyperlipidemic kidney injury

**Fig. 5:**
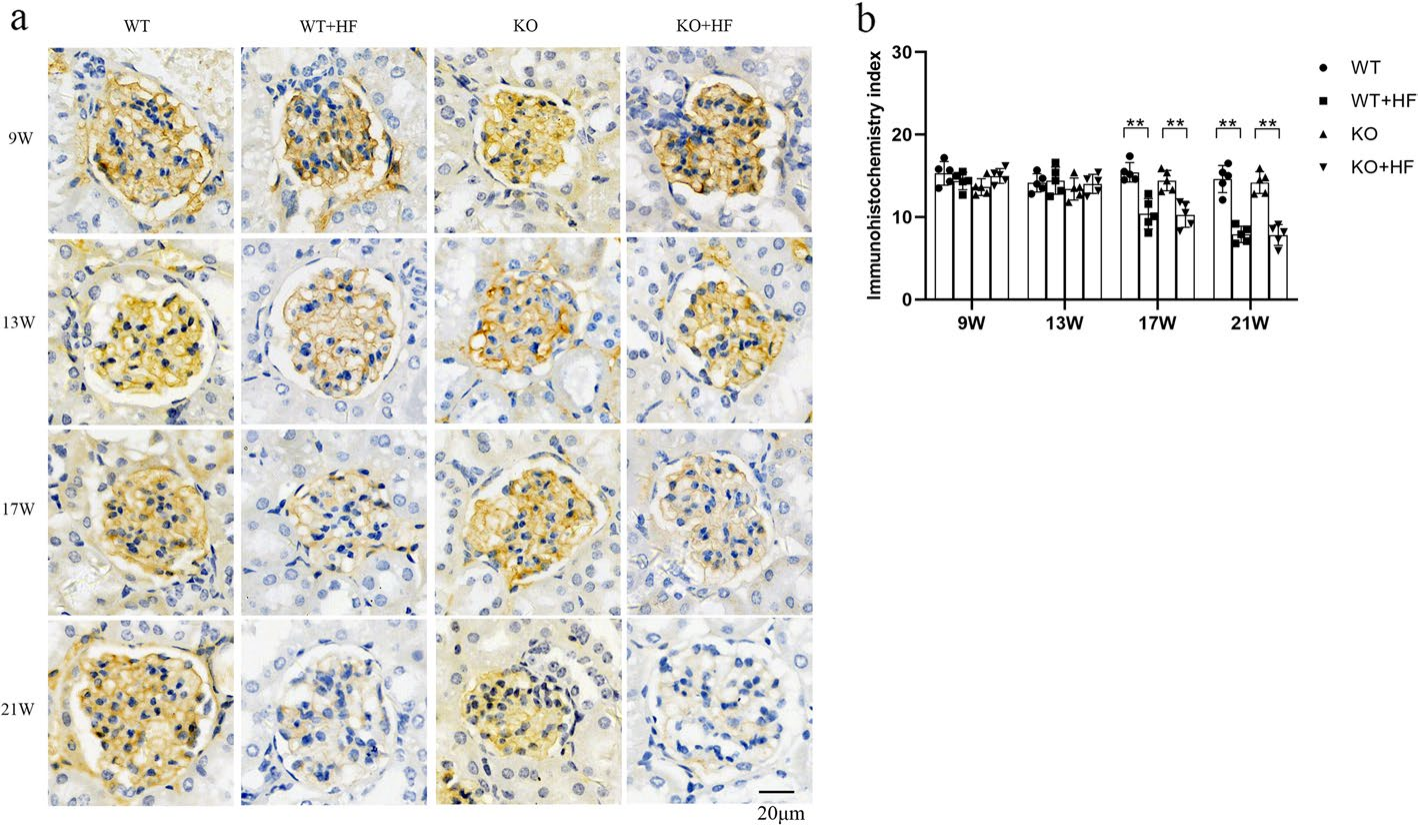
Lack of ANGPTL3 expression did not alleviate the podocin decline in podocytes in hyperlipidemic kidney injury. **a** The staining intensity of podocin is shown in the four groups; **b** podocin staining intensity gradually declined in both the WT + HF and KO + HF groups. (Bars = 20 μm)

Podocin has been demonstrated to be an important component of lipid rafts in podocytes. The disorder of cholesterol flow in lipid rafts will lead to the abnormal expression of podocin and then will affect the function of podocytes. In this study, the results showed that podocin staining decreased gradually in the WT + HF group (Fig. 5a). However, with prolonged modeling time, in WT + HF glomeruli, podocin expression weakened significantly at week 17 and was weaker than that in WT glomeruli, and this phenomenon was more obvious at week 21 (Fig. 5b).

We continued to explore the possible effect of *angptl3* knockout on podocin expression under normal physiological conditions. As shown in Fig. 5, the expression of podocin in the glomerulus of the KO group was close to that of the WT group at all four time points. Disappointingly, compared to expression in wild-type mice, podocin expression showed no improvement in the mice with knockout of the *angptl3* gene after a high-fat diet.

### CD2AP may not be involved in hyperlipidemic-related kidney injury

**Fig. 6:**
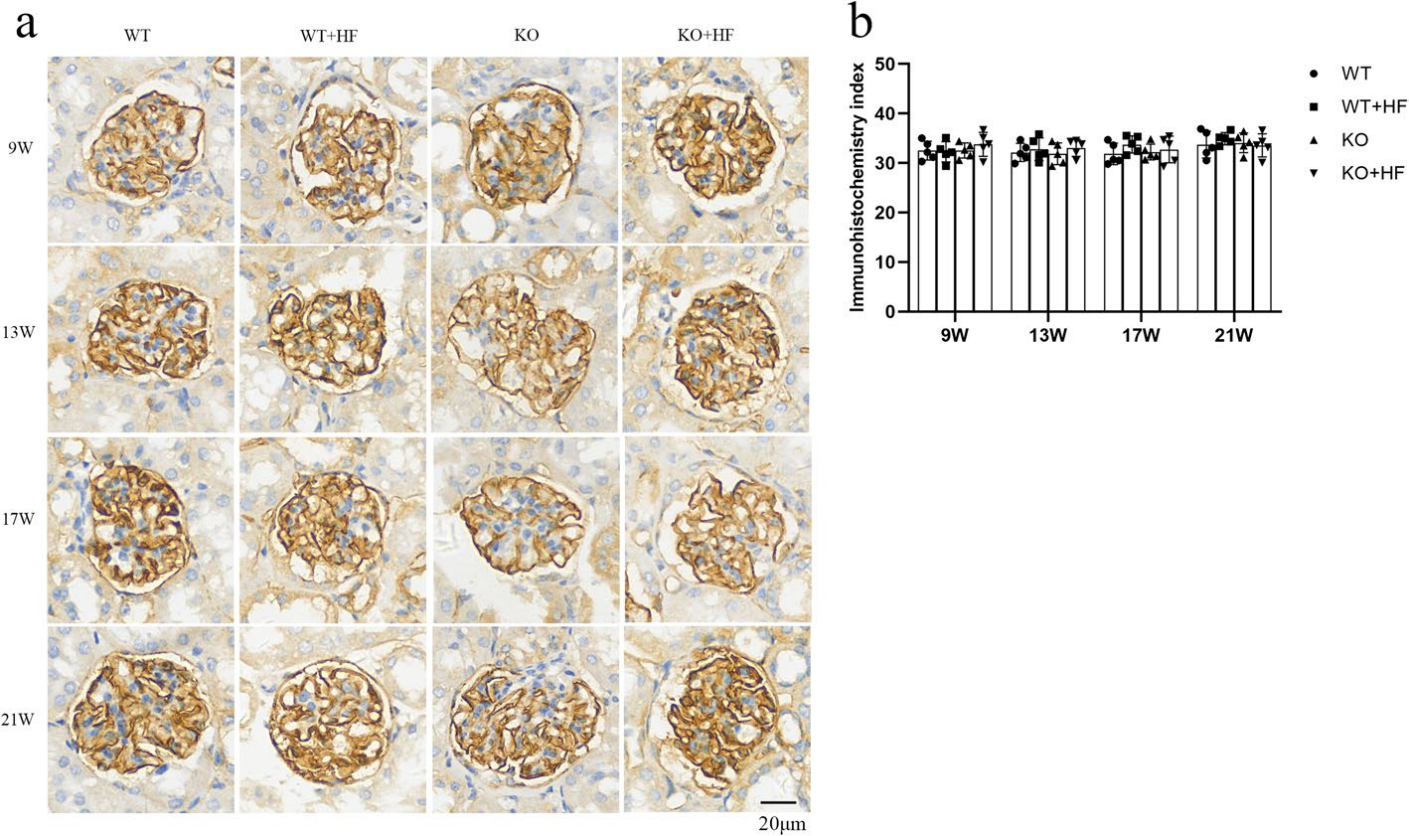
Expression of CD2AP was not affected after *angptl3* knockout. **a** The staining intensity of CD2AP is shown in the four groups; **b** the IHC index showed no significant difference among the groups. (Bars = 20 μm)

CD2AP is a solid molecule in the podocyte slit diaphragm. Apart from that, it has a signal correlation with podocin. High-fat feeding reduced the expression of podocin but did not affect CD2AP. The results are shown in Fig. 6. At each time point, CD2AP was not significantly altered in the wild-type group fed a high-fat diet. Under a normal diet, CD2AP expression between WT and KO group was similar. At the same time, we also did not find any difference in CD2AP expression in either mouse group after high-fat diet feeding. The intensity of immunohistochemical staining for CD2AP was comparable between the WT + HF and KO groups.

## Discussion

It is well known that injury to podocytes, the outermost layer of the glomerular filtration barrier, is the most common pathologic phenomenon and a dominant player in the disease progression of CKD. As highly differentiated epithelial cells, podocytes are connected to FPs by the slit diaphragm (SD). Recently, published research has shown that the SD is a special cholesterol-rich raft structure [12]. As an important component of the cell membrane, lipid rafts are involved in regulating cell membrane fluidity, membrane protein exchange, signal transduction and other functions [13]. Intracellular cholesterol homeostasis (such as the normal secretion, inflow and outflow of cholesterol in cells) is an important part of ensuring the normal function of cells [14]. To date, a great deal of progress has been made regarding the mechanism of podocyte lipid metabolism disorder.

ANGPTL3 is necessary to regulate the activity of LPL. More recent studies have demonstrated that this molecule is not only expressed in podocytes but is also related to podocyte injury, such as increased podocyte motility and FPs fusion [15]. In the previous study, we reported that serum ANGPTL3 was significantly enhanced in patients with hyperlipidemia-related kidney injury [6]. In this study, the data further confirm that glomerular ANGPTL3 expression is enhanced in a mouse model of renal injury associated with hyperlipidemia. Recently, we also reported that Angptl3 transgenic mice exhibited hyperlipidemia and proteinuria, and TEM showed that the podocyte FPs were effaced [11]. In this hyperlipidemia-related renal injury mouse model, with prolonged high-fat feeding time, the expression of ANGPTL3 gradually increased, and podocyte FPs fusion was increased accordingly. Importantly, compared with the wild-type mice, in the mice without the *angptl3* gene that were fed the same high-fat diet, the serum lipids did not increase significantly, proteinuria also obviously recovered, and the severity of podocyte FPs fusion was prominently relieved.

These results suggested that ANGPTL3 not only affects hyperlipidemia induced by a high-fat diet but also participates in renal injury in this model. ANGPTL3 may be involved in the glomerular injury induced by hyperlipidemia. Currently, inhibitors of ANGPTL3 have been extensively studied in cardiovascular disease, and suppression of ANGPTL3 is protective in nephrotic syndrome [16, 17]; in the future, this inhibitor would be a candidate for hyperlipidemia-related kidney injury.

Podocytes are terminally differentiated epithelial cells with abundant cytoskeletal proteins. The rearrangement of cytoskeletal actin includes the polarity loss of the arrangement of stress filaments and the abnormal expression of cytoskeletal proteins. At present, it is generally believed that cytoskeletal actin rearrangement is the molecular basis of podocyte motility. When rearrangement occurs, podocyte junctions are weakened, and podocyte adhesion is lost [18]. The loss of podocyte adhesion is an important pathological basis of glomerulosclerosis.

Following the widespread clinical use of second-generation sequencing technology, the *actn4* gene mutation was found in patients who suffered from congenital kidney disease. The abnormality of the *actn4* gene led to the occurrence of steroid-resistant congenital kidney disease. The pathological type was FSGS [19]. Recent published studies have shown that podocytes with homozygous mutation of the *actn4* gene may have an irrecoverable decrease in intercellular connectivity and the occurrence of cytoskeleton rearrangement [20]. In our published studies, we also confirmed that ANGPTL3 can affect the expression levels of podocyte lipid raft-associated molecules such as nephrin and ACTN4 in an adriamycin-induced nephropathy model and can then aggravate the cytoskeleton rearrangement and cell motility of podocytes [15, 21]. ANGPTL3 can play a role through ACTN4 in a hyperlipidemia-related kidney injury model has not been reported.

The IHC data primarily revealed that the ACTN4 staining intensity was weakened in hyperlipidemia-related glomeruli with prolonged high-fat diet feeding, suggesting that ACTN4 may be involved in podocyte injury caused by hyperlipidemia. After gene knockout of *angptl3*, the decrease in ACTN4 was significantly inhibited in the glomeruli of mice treat with high-fat diet, suggesting that ACTN4 is a potential molecular signal in ANGPTL3’s mechanism for hyperlipidemia-related renal injury. ACTN4 is a potential signaling molecule for ANGPTL3 in the mechanism of hyperlipidemia-related kidney injury. ANGPTL3 can bind to and activate the podocyte integrin β3 receptor, phosphorylate FAK, and ultimately change ACTN4 expression in ADR-treated podocytes in vitro [7]*.* However, how ANGPTL3 impacts ACTN4 in podocyte injury induced by high fat has not been well clarified.

Currently, so many studies have reported that mutations in the NPHS2 gene can trigger congenital kidney disease, and the protein podocin encoded by the NPHS2 gene is an intrinsic molecule of the podocyte SD. Schermer B et al. found that podocin must bind with cholesterol to activate transient receptor potential channel 6 and then complete signal transduction and participate in podocyte function regulation [22]. This study discovered that a high-fat diet induced a noteworthy fading of podocin staining intensity in wild-type glomeruli. However, without the *angptl3* gene did not reverse the fate of the podocin decline after a high-fat diet. This means that podocin might not be a candidate for the ANGPTL3 mechanism in lipid-related podocyte injury.

As an adapter molecule, CD2AP could contact the cytoplasmic domain of nephrin and form the podocyte SD [23]. CD2AP is related to cytoskeletal actin rearrangement via the interplay between nephrin and the actin cytoskeleton [24]. Unexpectedly, we did not find any significant changes in glomerular CD2AP expression in WT + HF mice. We also did not find any differences in glomerular CD2AP expression in the mice with knockout of the *angptl3* gene with or without a high-fat diet. There were no obvious changes in CD2AP expression in glomeruli between wild-type and *angptl3* knockout mice. Therefore, we think that the reactions between various podocyte molecules are quite complex [25]. The interactions between some podocyte molecules need further exploration.

Finally, mice after high-fat feeding, the degree of glomerular injury was slight, and only podocyte FPs fusion occurred. The biochemical and pathological changes in the model were only observed within 21 weeks. Prolonging the observation time is necessary to further understand the kidney injury in this model.

In the later stage of mice on a high-fat diet, renal function gradually deteriorated, accompanied by a gradual decrease in ACTN4 in the kidney. However, after knockdown of the *angptl3* gene, ACTN4 was not decreased, indicating that there might be some interaction between them and that ANGPTL3 promoted the expression of ACTN4 or inhibited the degradation of ACTN4 in some manner.

### Study strength and limitations

This study has several strengths. WT and ANGPTL3 knockout mice were divided into groups based on a normal diet or a high-fat diet. Renal tissue was taken for pathological evaluation by H&E and transmission electron microscopy. Ultrastructural results showed that knockout of the *angptl3* gene could alleviate podocyte damage in mice caused by high-fat feeding. In addition, immunohistochemistry staining showed that the decrease in ACTN4 expression is related to podocyte injury in mice. There are also some limitations. ANGPTL3 and ACTN4 are involved in podocyte injury, but the pathways of ANGPTL3 and ACTN4 in glomerular podocyte injury and the specific relationship between these two proteins are not explained in detail.

## Conclusions

In summary, our study suggests that ANGPTL3 may participate in renal injury in hyperlipidemia and that ACTN4 is its target molecule in this mechanism. In patients suffering from hyperlipidemia and impaired renal function, inhibitors of ANGPTL3 were used to reduce serum lipids, thereby improving the renal function of the patient to improve the function of the kidney. ANGPTL3 inhibitors may also be a drug for the treatment of this disease.

## Supplementary Information

**Additional file 1.**

**Additional file 2.**

**Additional file 3.**

**Additional file 4.**

## Data Availability

All data generated during this study are included in this published article.

## Abbreviations

ANGPTL3: Angiopoietin-like 3
WT: Wild-type with normal diet
KO: *angptl3*-/- With normal diet
WT + HF: Wild-type + high-fat diet
KO + HF: *angptl3*-/- + High-fat diet
IHC: Immunohistochemistry
FPs: Foot process
LPL: Lipoprotein lipase
TG: Triglyceride
TC: Total cholesterol
CKD: Chronic kidney disease
ACTN4: Alpha-Actinin-4
CD2AP: CD2 associated protein
FSGS: Focal segmental glomerulosclerosis
SD: Slit diaphragm
