# Supplementary material for: ANGPTL3 is involved in kidney injury in high-fat diet-fed mice by suppressing ACTN4 expression
Source: Lipids Health Dis. 2022 Sep 19;21:90. doi: 10.1186/s12944-022-01700-3 (PMC9487085; doi:10.1186/s12944-022-01700-3)
Supplement: Supplementary file 1 — Additional file 1. [file 12944_2022_1700_MOESM1_ESM.pptx]

## Slide 1
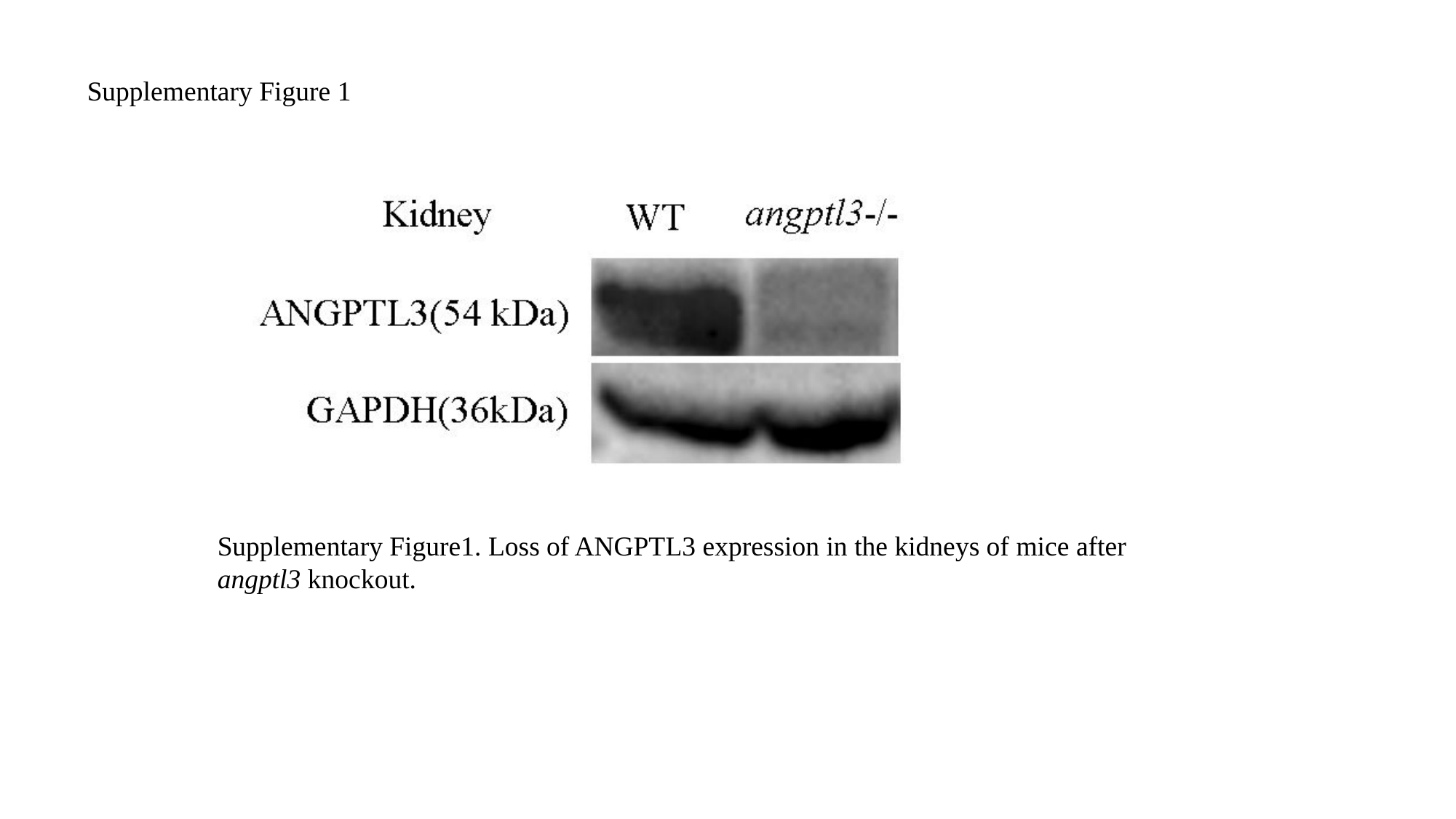

Supplementary Figure 1
Supplementary Figure1. Loss of ANGPTL3 expression in the kidneys of mice after angptl3 knockout.

## Slide 2
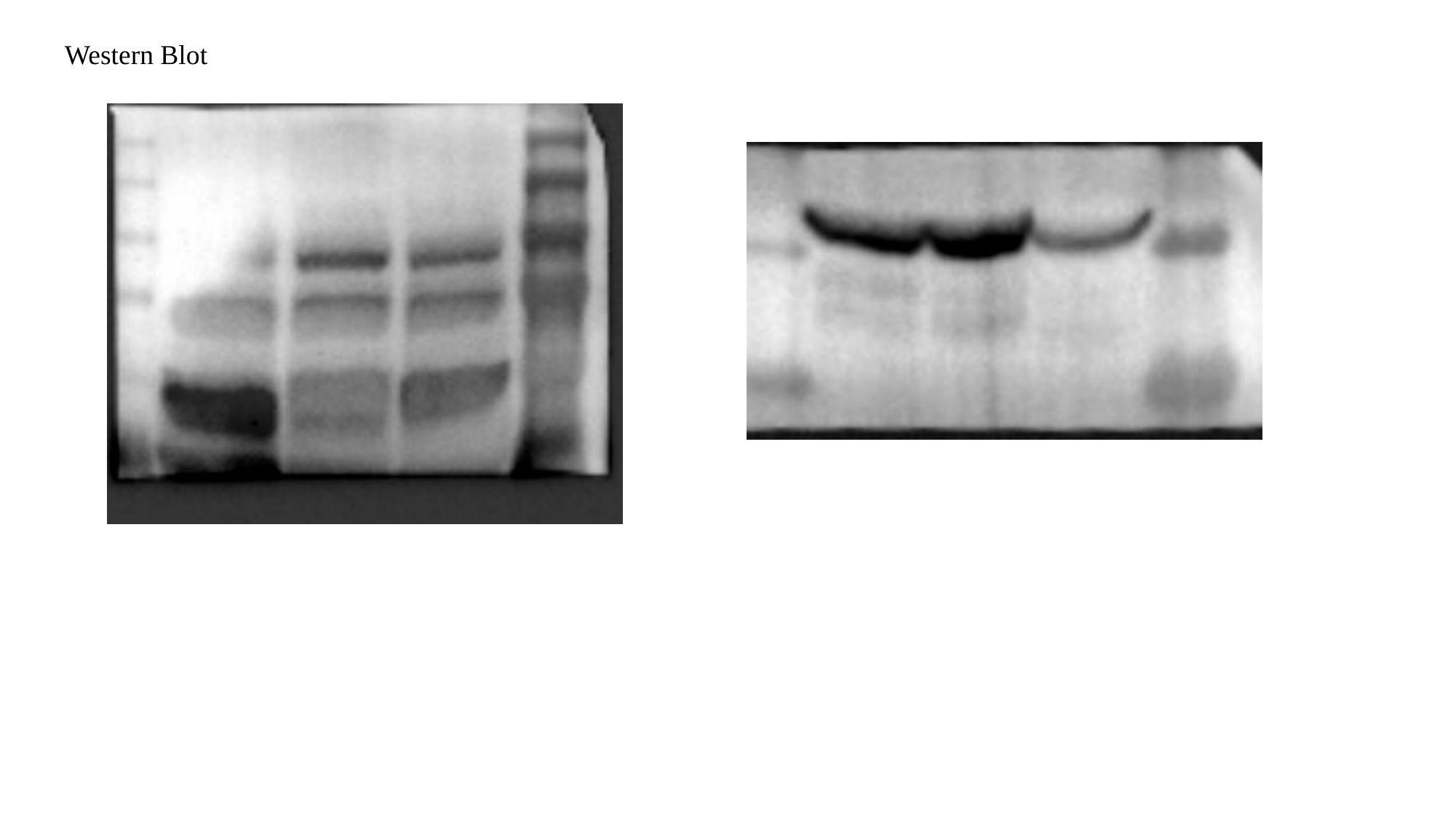

Western Blot
