# Supplementary figures and images for: ANGPTL3 is involved in kidney injury in high-fat diet-fed mice by suppressing ACTN4 expression
Source: Lipids Health Dis. 2022 Sep 19;21:90. doi: 10.1186/s12944-022-01700-3 (PMC9487085; doi:10.1186/s12944-022-01700-3)

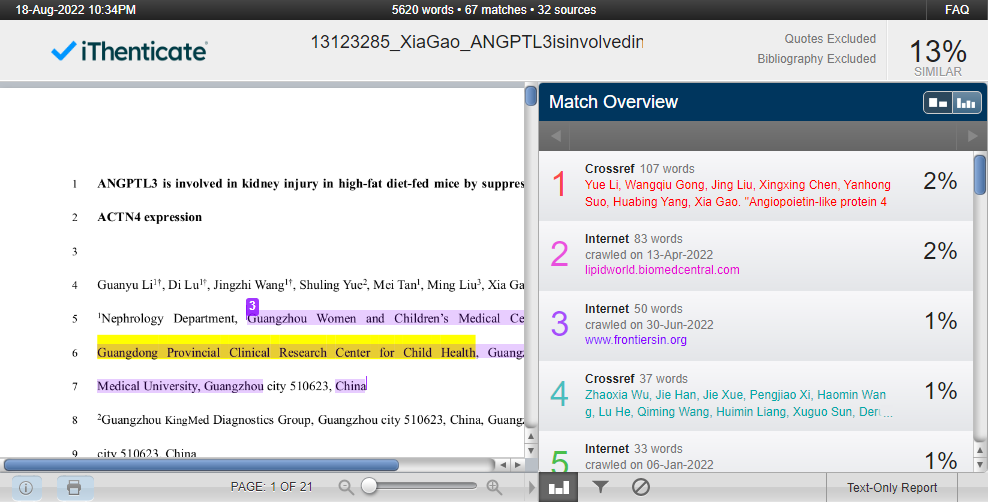

Supplement: Supplementary file 3 — Additional file 3. [file 12944_2022_1700_MOESM3_ESM.png]
