# Supplementary material for: ANGPTL3 is involved in kidney injury in high-fat diet-fed mice by suppressing ACTN4 expression
Source: Lipids Health Dis. 2022 Sep 19;21:90. doi: 10.1186/s12944-022-01700-3 (PMC9487085; doi:10.1186/s12944-022-01700-3)
Supplement: Supplementary file 4 — Additional file 4. [file 12944_2022_1700_MOESM4_ESM.pdf]

SPRINGER NATURE
Author Services

Editing Certificate

This document certifies that the manuscript

ANGPTL3 is involved in kidney injury in high-fat diet mice by suppressing ACTN4 expression

prepared by the authors

Xia Gao

was edited for proper English language, grammar, punctuation, spelling, and overall style by one or more of the highly qualified native English speaking editors at SNAS.

This certificate was issued on **August 15, 2022** and may be verified on the SNAS website using the verification code **8D44-EE94-CBC9-0441-1040**.

Neither the research content nor the authors' intentions were altered in any way during the editing process. Documents receiving this certification should be English-ready for publication; however, the author has the ability to accept or reject our suggestions and changes. To verify the final SNAS edited version, please visit our verification page at secure.authorservices.springernature.com/certificate/verify. If you have any questions or concerns about this edited document, please contact SNAS at support@as.springernature.com.

SNAS provides a range of editing, translation, and manuscript services for researchers and publishers around the world. For more information about our company, services, and partner discounts, please visit authorservices.springernature.com.
